# Recombinant pre-miR-29b for Alzheimer´s disease therapeutics

**DOI:** 10.1038/srep19946

**Published:** 2016-01-28

**Authors:** Patrícia A. Pereira, Joana F. Tomás, João A. Queiroz, Ana R. Figueiras, Fani Sousa

**Affiliations:** 1 CICS-UBI – Health Sciences Research Centre, University of Beira Interior, Avenida Infante D. Henrique, Covilhã, 6200-506, Portugal; 2 CNC – Center of Neuroscience and Cell Biology, University of Coimbra, Largo Marquês de Pombal, Coimbra, 3004-517, Portugal

## Abstract

MicroRNAs are arising as the next generation of diagnostic and therapeutic tools for gene silencing. Studies demonstrated that the miR-29 expression is decreased in Alzheimer’s disease (AD) patients displaying high levels of human β-secretase (hBACE1). Recent advances toward an effective therapy for AD intend to employ miR-29 to suppress hBACE1 expression and subsequent Amyloid-β (Aβ) peptide. However, delivery of mature miRNA has demonstrated modest efficacy *in vitro*; therefore, the preparation of highly pure and biologically active pre-miRNA arises as one of the most important challenges in the development of these therapeutic strategies. Recently, we described a new strategy based arginine-affinity chromatography to specifically purify the recombinant pre-miR-29b. Following this strategy, the purified pre-miR-29b was successfully encapsulated into polyplexes that were further delivered in cytoplasm. It was verified that Chitosan/pre-miR-29b and Polyethylenimine/pre-miR-29b systems efficiently delivered pre-miR-29b to N2a695 cells, thus reducing the hBACE1 protein expression (around 78% and 86%, respectively) and Aβ_42_ levels (approximately 44% and 47%, respectively). Furthermore, pre-miR-29b downregulates the hBACE1 mRNA expression in 80%. Overall, it was demonstrated that the recombinant pre-miR-29b using polyplexes allowed to decrease the hBACE1 and Aβ_42_ expression levels, improving the currently available methodologies of miRNA-based therapeutics.

Over the past decade, several hundred small non-coding regulatory RNA molecules, known as microRNAs (miRNAs), have been identified as potential biomarkers and therapeutic products^1^,^2^. Briefly, miRNAs are initially transcribed as pri-miRNAs from the regions of the genome that are subsequently processed by the Drosha into pre-miRNAs stem-loop. Then, pre-miRNAs are exported to the cytoplasm where they are converted into mature miRNAs after the removal of their loops by Dicer. Finally, one strand of the miRNA duplex (called mature miRNA sequence) is loaded onto the RNA-induced silencing complex (RISC) and, posteriorly, binds to specific sites typically present in the 3′ untranslated region (3′UTR) of messenger RNA (mRNA), leading to translational inhibition with imperfect base pairing or, less frequently, to the mRNA degradation with perfect base pairing^1^,^3^,^4^,^5^,^6^. An important feature of these RNAs is that an individual miRNA can regulate multiple mRNA targets and, consequently, can induce multiple effects on the expression of several related genes families^3^. Accordingly, it is likely that miRNAs are directly involved in a variety of cellular processes, namely in cell cycle regulation, proliferation, apoptosis, differentiation, stress response and metabolism^5^,^6^,^7^,^8^,^9^. Thus, the unveiling of the role of several miRNAs present in cellular processes is critical to develop new diagnosis methods or drugs for understanding and treating illnesses. To date, there are over 1880 miRNAs that have been discovered in humans, among which the miR-29 remains as one of the most interesting and intriguing miRNA families, once the dysregulation of this miRNA has a strong impact on many diseases including multiple types of cancers^10^,^11^,^12^,^13^,^14^ and neurodegenerative^15^,^16^,^17^,^18^,^19^ disorders, suggesting that it can be a promising target in the therapeutic of these diseases.

Recently, several studies have been published concerning the expression of miR-29 in patients with Alzheimer’s disease (AD)^16^,^20^,^21^, suggesting that this biomolecule can play an important role in the regulation of this neurodegenerative disease. The generation and subsequent accumulation of amyloid-β peptides (Aβ) is a histological characteristic that is strongly implicated in the pathogenesis of AD^22^,^23^,^24^. One of the mechanisms that contribute to the accumulation of Aβ is sequential proteolytic cleavages of full-length amyloid precursor protein (APP) by β-site amyloid precursor protein cleaving enzyme 1 (BACE1), being considered as a prime drug target for therapeutic inhibition of Aβ production in AD^25^. Hence, the regulation of the proteins expression levels involved in the Aβ generation process has demonstrated to be important in AD. Several research groups showed that the miR-29 is potentially involved in the regulation of APP and BACE1 expression because *in vitro* studies revealed that in sporadic AD patients, displaying abnormally high BACE1 protein levels, the miR-29 cluster was significantly decreased^16^,^19^,^21^. These findings provided support for a causal relationship between miR-29 and AD, since the miR-29b suppresses the expression levels of BACE1 and, consequently, Aβ peptides levels in neuronal cells^16^. Thus, miR-29 can be used as a potential therapeutic weapon for pharmacological intervention of AD.

Commonly, the miRNAs used for therapeutic purposes have mostly been produced by chemical synthesis (phosphoamidite chemistry, normally used for the generation of short oligoribonucleotides), enzymatic synthesis (longer RNAs can be produced by *in vitro* transcription) or via a plasmid (for miRNA expression into eukaryotic cell lines)^26^. Although these methods can be very efficient in producing miRNA, in general, additional purification protocols to remove the impurities (failure sequences, impurities of pDNA template, enzymes, nucleotides, salts or buffer) from the production process have to be employed. The presence of impurities may lead to non-targeted gene silencing, what is commonly associated with a decrease in therapeutic effectiveness and still restrict the implementation of these oligonucleotides onto clinical applications^26^,^27^. Furthermore, the purification methods usually employed are still expensive, difficult to scale up and can cause degradation of the RNA molecules due to the requirement of toxic solvents and use of denaturing conditions^28^. Thus, considering the rapidly growing interest on these novel biopharmaceuticals, as a result of its potential therapeutic application, novel technologies to improve their preparation are currently being pursued.

Thus, our research group recently proposed a new process for production and purification through amino acid-based affinity chromatography, of recombinant pre-miR-29b with potential therapeutic application^29^. This strategy could contribute for the establishment of reliable, simple and more cost-effective processes, easily adopted by biopharmaceutical industries, while maintaining maximal product quality and biological activity. However, in order to improve the biological effect in RNAi-based therapies it is also essential the development of an efficient miRNA delivery system capable to overcome the biological barriers, protect the integrity of miRNA and trapping miRNA in intracellular space to exert its function^30^. During the last decades, several research groups have focused on the production and characterization of polyplexes formed between sRNA and some cationic polymers such as Polyethylenimine (PEI)^31^,^32^,^33^,^34^,^35^ and Chitosan (CS)^36^,^37^,^38^,^39^,^40^. In general, these vehicles can be promising systems to compact RNA for systemic delivery because they present a mean diameter of 200 nm, high stability, biocompatibility and proved to be useful in protecting the RNA against fetal bovine serum (FBS) and ribonuclease^41^. Furthermore, delivery by polyplexes has the advantage of being more cost-effective. However, the high toxicity of PEI is one of the major limiting factors especially for *in vivo* use. Polymers with low molecular weight (<25 KDa) display low toxicity, but the transfection efficiency is low as well. It is commonly believed that the most suitable molecular weight of PEI for gene transfer ranges between 5KDa and 25KDa^42^,^43^.

Thus, the main novelty of the present study is to report the application of the recombinant pre-miR-29b in cells transfection to decrease the hBACE1 expression levels and, subsequently the Aβ expression levels. Overall, the implementation of this cutting-edge approach provides the basis for the improvement of the currently available methodologies of gene silencing as a putative genetic therapy for AD and their implementation on biopharmaceutical industry.

## Results

### pre-miR-29b protection by polyplexes formulation and delivery to neuronal cells

**Figure 1:**
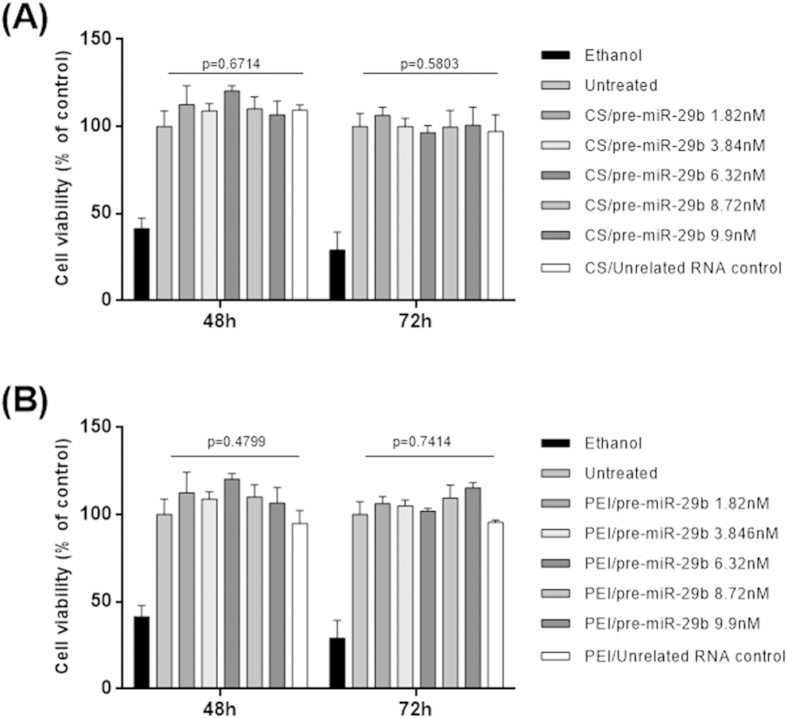
MTS assays conducted in CS/pre-miR-29b (A) and in PEI/pre-miR-29b (B), which were cultured in the presence of increasing concentrations of pre-miR-29b for 48 and 72 h. Untreated cells and cells transfected with an unrelated RNA control were used as negative controls for cytotoxicity. Ethanol treated cells were used as positive control to induce toxicity. Mean percentage values relative to the untreated cells and standard error of the mean in 3 independent experiments are shown. ANOVA, mean ± SD.

The pre-miR-29 loaded polyplexes were formulated using the following conditions: CS/pre-miR-29b with N/P ratio of 30 and to PEI/pre-miR-29b with N/P ratio of 3.5 (see Methods section). Relevant parameters such as size, zeta potential and loading capacity were determined for the different polyplexes. As shown in Table 1, all of the polyplexes demonstrated high encapsulation efficiency, small sizes and exhibited a strong positive charge on their surface. In addition, the cellular cytotoxicity effect of all the synthesized formulations (polyplexes/pre-miR-29b) was evaluated by MTS and compared with cells treated with ethanol (positive control). As presented in Fig. 1, at 48 and 72 h after transfection, cellular viability is clearly not affected by the presence of the CS/pre-miR-29b and PEI/pre-miR-29b since the majority of cells remained viable (>94% viability), suggesting that these carriers are suitable for therapeutic applications.

### Downregulation of human BACE1 expression induced by polyplex/pre-miR-29b

**Figure 2:**
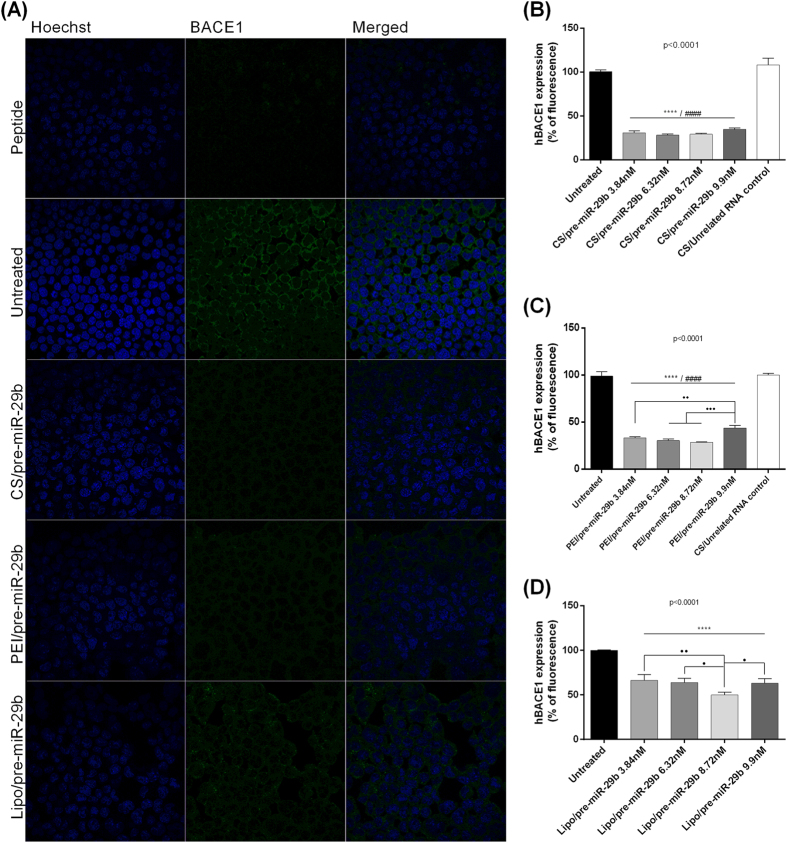
Recombinant pre-miR-29b effect on BACE1 levels in N2a695 cells at different concentrations of the pre-miR-29b for 72 h. **(A)** Representative confocal microscopy images of N2a695 cells treated with CS/pre-miR-29b, PEI/pre-miR-29b and Lipo/pre-miR-29b stained against BACE1. Quantification of fluorescence intensity for BACE1 protein expression in cells transfected with: **(B)** CS/pre-miR-29b, **(C)** PEI/pre-miR-29b, and **(D)** Lipo/pre-miR-29b. All results are expressed relatively to those in untreated cells and error bars represent standard deviations derived from three or more independent experiments performed in triplicate. ANOVA, mean ± SD.

**Figure 3:**
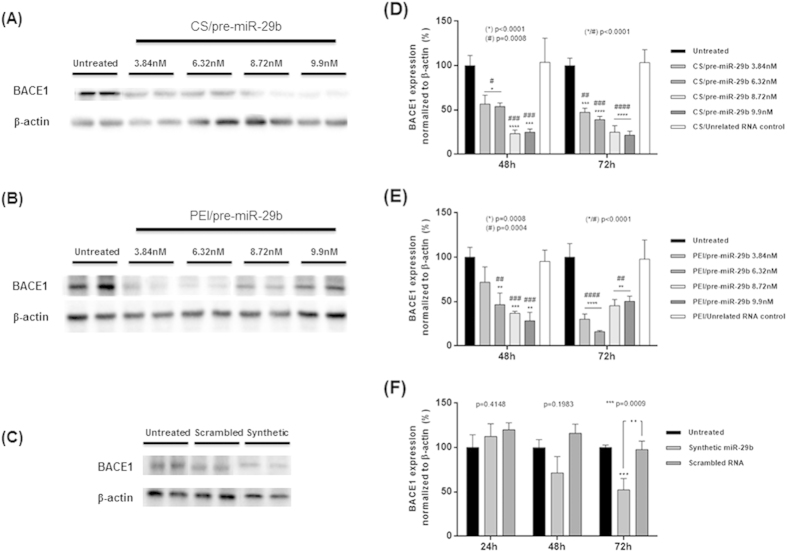
Western blot analysis of endogenous hBACE1 and β-actin levels in the cell lysate of N2a695 cells treated with different concentrations of pre-miR-29b, at 24, 48 and 72 h. **(A–C)**: representative images of Western blot from N2a695 cells after 72 h transfection with CS/pre-miR-29b, PEI/pre-miR-29b and Synthetic miR-29b and Scrambled RNA, respectively. **(D–F)**: densitometric quantifications of hBACE1 expression from Western blot images of CS/pre-miR-29b, PEI/pre-miR-29b and Synthetic miR-29b and Scrambled RNA, respectively. Error bars represent standard deviations derived from three or more independent experiments performed in duplicate. ANOVA, mean ± SD. (For simplicity purposes, the blots were cropped).

To ascertain whether recombinant pre-miR-29b could effectively suppress human BACE1 (hBACE1) expression, we used mouse neuroblastoma (N2a) cells stably transfected with cDNAs encoding human APP695 (N2a695 cells)^44^. Thus, in order to explore the effect of recombinant pre-miR-29b administration, N2a695 cells were transfected with CS/pre-miR-29b, PEI/pre-miR-29b and Lipo/pre-miR-29b using different concentrations of the pre-miR-29b (3.84, 6.32, 8.72 and 9.9 nM). Consistent with the Immunocytochemistry results, Western blot analysis revealed that endogenous hBACE1 protein levels were significantly reduced in the N2a695 cell line transfected with polyplexes containing the recombinant pre-miR-29b, compared with the untreated cells and cells transfected with an unrelated RNA control (Figs 2 and 3), at 48 and 72 h. In addition, N2a695 cells were transfected with synthetic miR-29b (positive control) and scrambled RNA (negative control) (at a final concentration of 9.9 nM) (Fig. 3F). At 24 h after transfection, no significant changes on the endogenous levels of hBACE1 protein were detected when compared with untreated cells and cells transfected with the unrelated RNA control (data not shown). On the other hand, 72 h after transfection, hBACE1 protein expression was decreased by approximately 78% in cells transfected with CS/pre-miR-29b, and by 86% in those transfected by PEI/pre-miR-29b complexes (Figs 2 and 3). In addition, in N2a695 cells, pre-miR-29b treatment caused concentration-dependent inhibition of hBACE1 expression (Fig. 3). Thus, hBACE1 protein expression was significantly reduced following transfection with both synthetic miR-29b (around 48% reduction) and recombinant pre-miR-29b (around 82% reduction) by comparing with the transfection of untreated cells (Fig. 3C). Therefore, endogenous hBACE1 levels are significantly inhibited by pre-miR-29b delivery in N2a695. To confirm the antibody specificity, the primary antibody anti-BACE1 was blocked with BACE1 peptide, by incubation at RT during 4 h. As demonstrated by Fig. 2A, fluorescence was not detected in the confocal microscopy image, indicating that the antibody binds to BACE1 in N2a695 cells, proving the high specificity of antibody to hBACE1.

### Pre-miR-29b inhibits hBACE1 translation and affect hBACE1 mRNA level

**Figure 4:**
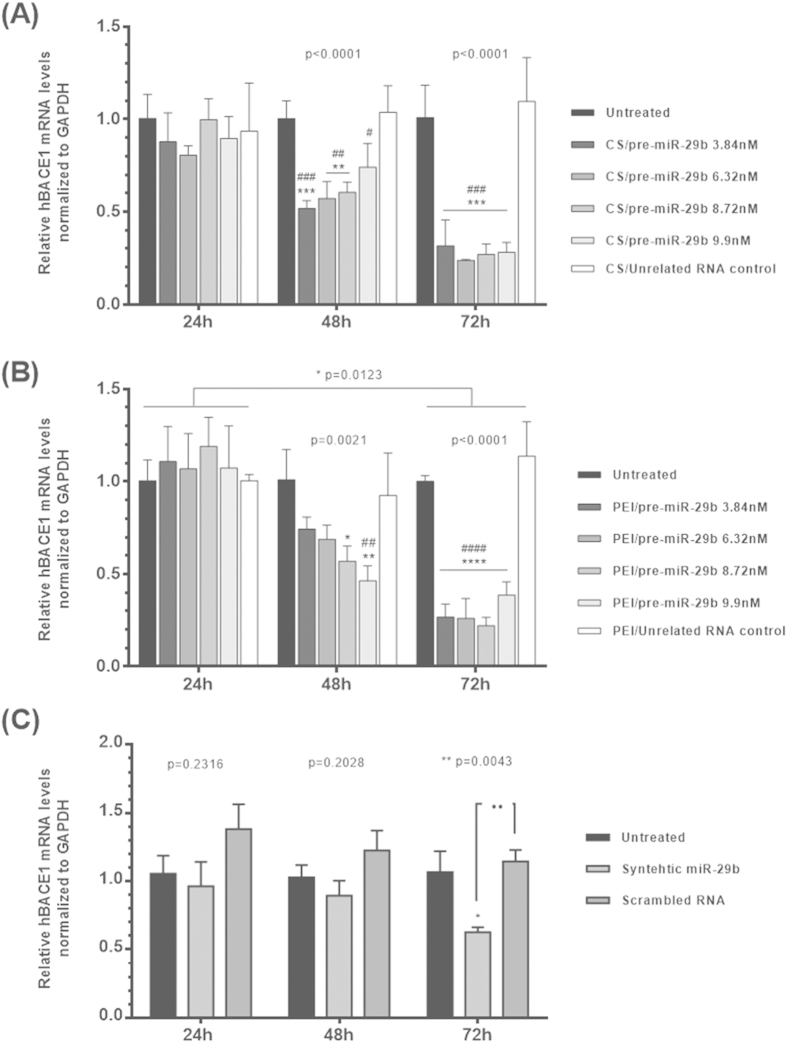
Effect of recombinant pre-miR-29b on hBACE1 mRNA levels in N2a695 cells following 24, 48 and 72 h treatment with: (A) CS/pre-miR-29b and (B) PEI/pre-miR-29b (C) Synthetic miR-29b and Scrambled RNA. Values in the graphs are mean from triplicates of RT-qPCR threshold cycles for hBACE1 mRNA normalized to those of mRNA for GAPDH from 3 independent experiments and demonstrate significant differences across treatment conditions. ANOVA, mean ± SD.

Following the evaluation of the effect of the transfection of N2a695 cells with polyplexes loaded with recombinant pre-miR-29b on hBACE1 protein levels through Immunocytochemistry and Western-blot analysis, it was also evaluated its effect on hBACE1 mRNA levels by RT-qPCR measurements. As expected, RT-qPCR results (Fig. 4) revealed that hBACE1 mRNA levels in N2a695 cells were also significantly reduced after treatment with CS/pre-miR-29b and PEI/pre-miR-29b relatively to untreated cells and cells transfected with the unrelated RNA control (Fig. 4). As a matter of fact, miRNAs can regulate gene expression through either translational suppression, mRNA degradation or both. Data analysis showed that CS/pre-miR-29b 6.32 nM significantly decreased hBACE1 mRNA expression to 76.4 ± 0.6% (Fig. 4A) relatively to N2a695 cells treated with PEI/pre-miR-29b 8.72 nM (77.9 ± 4.5%), at 72 h of transfection (Fig. 4B). At 48 h after transfection, a significant reduction of hBACE1 mRNA levels was also verified, being 48.5 ± 4.4% and 53.8 ± 8.1%, respectively for CS/pre-miR-29b 3.84 nM and PEI/pre-miR-29b 9.9 nM (Figs 4A,B). Furthermore, it was also performed RT-qPCR for RNA from N2a695 cells transfected with scrambled RNA or with synthetic miR-29b, at 24, 48 and 72 h (Fig. 4C). As expected, hBACE1 mRNA levels were also decreased in comparison with untreated control and cells transfected with an unrelated RNA control, in the cells transfected with synthetic miR-29b (41.3 ± 3.6%), at 72 h (Fig. 4C). On the other hand, hBACE1 mRNA levels were unaffected in cells transfected with scrambled RNA (Fig. 4C). Thus, the decrease in hBACE1 protein levels is likely due to a decrease in hBACE1 mRNA stability or by reducing the transcription rate since we found by RT-qPCR that the level of hBACE1 mRNA was also decreased by recombinant pre-miR-29b and synthetic miR-29b delivery (Fig. 4)^45^. Furthermore, the correlation between hBACE1expression and pre-miR-29b provides further support for the hypothesis that pre-miR-29b contributes, at least in part, to overall changes in hBACE1 expression in AD. Taken together, these data demonstrate effective delivery of recombinant pre-miR-29b to N2a695 cells using polyplexes and that a specific suppression of hBACE1 expression occurs when these polyplexes delivered pre-miR-29b.

### Recombinant pre-miR-29b modulates Aβ_42_ generation *in vitro*

**Figure 5:**
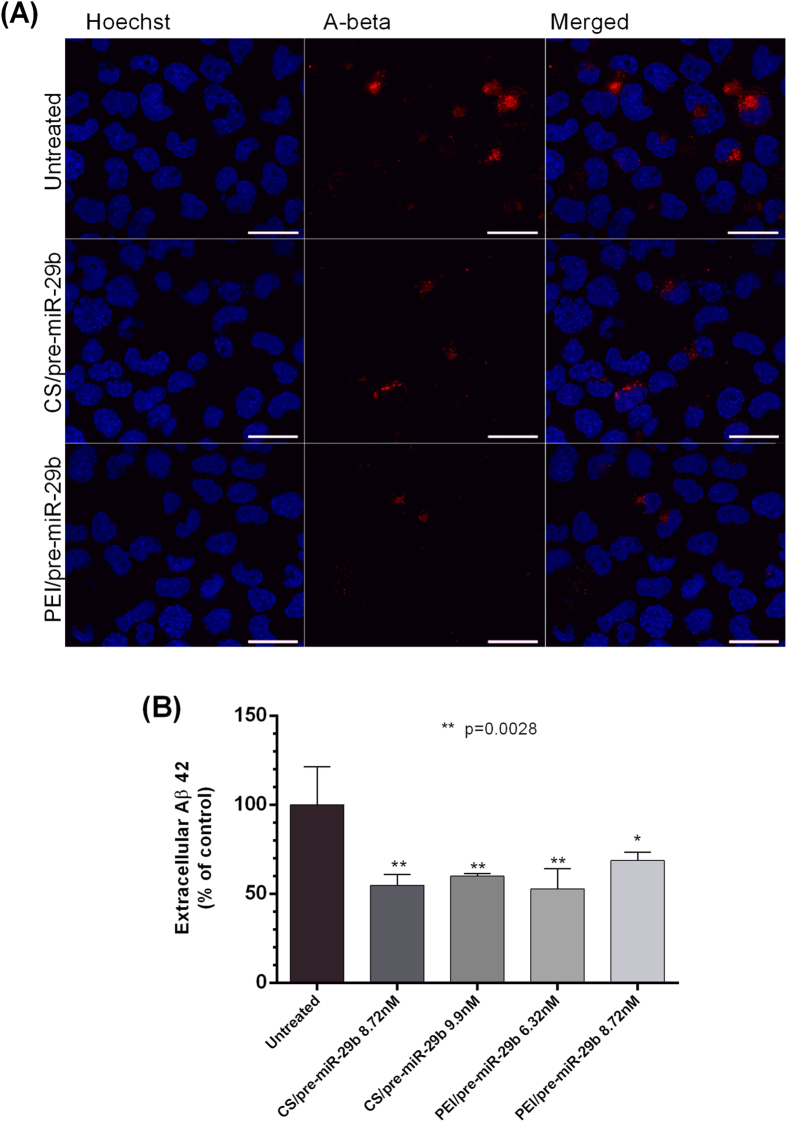
Effect of recombinant pre-miR-29b on modulation of Aβ levels in N2a695 cells. **(A)** Representative confocal microscopy images of untreated N2a695 cells and cells transfected with CS/pre-miR-29b and PEI/pre-miR-29b stained against Aβ_total_ peptide (scale bars 20 μm). **(B)** Aβ_42_ ELISA in cell lysates collected 72 h after transfection with CS/pre-miR-29b and PEI/pre-miR-29b. All results are expressed relative to those in untreated cells and presented as mean values from at least 3 independent experiments. ANOVA, mean ± SD.

Previous studies showed a causal relationship between miR-29b expression and BACE1 activity, and, consequently, Aβ peptides generation^16^. Interestingly, by Immunocytochemistry analysis it was found that when the endogenous Aβ_total_ peptides are secreted, they accumulate around the nucleus leading to its deformation, appearing like a kidney (Fig. 5A). Thus, to directly examine whether recombinant pre-miR-29b also reduces endogenous Aβ_42_ levels, the N2a695 cells (Fig. 5A) were transfected according to the best transfection conditions mentioned above (CS/pre-miR-29b and PEI/pre-miR-29b at 8.72/9.9 and 6.32/8.72 nM, respectively) to reduce the expression of hBACE1. Aβ_42_ levels in culture medium and in cell lysates were analyzed using ELISA (Fig. 5B). However, it was verified that Aβ_42_ could only be detected in medium but was absent in cell lysate of the transfected cells. As a result, endogenous Aβ_42_ levels were significantly reduced by 45.2 ± 6.0% and 40.07 ± 1.3% with CS/pre-miR-29b 8.72 and 9.9 nM, respectively. With PEI/pre-miR-29b 6.32 and 8.72 nM, the Aβ_42_ endogenous levels were reduced by 47.2 ± 11.4% and 31.31 ± 4.7%, respectively, when compared to N2a695 untreated cells at 72 h (Fig. 5B). Therefore, endogenous Aβ_42_ levels were inhibited by recombinant pre-miR-29b in this neuronal cell line. Once again, these findings indicate that recombinant pre-miR-29b regulates Aβ_42_ production through mechanisms dependent of hBACE1 expression.

## Discussion

BACE1 and Aβ are central players in the pathways implicated in AD, it has been established that reducing BACE1 expression has been suggested as a potential strategy for mitigating the pathological processes underlying AD^25^,^45^. Interestingly, the miR-29a/b-1/c cluster is significantly decreased in the brains of AD patients, and this decrease is correlated with the increased level of BACE1 protein^16^,^21^. Previously published studies have demonstrated the importance of using the miR-29 family as a potential major suppressor to silence BACE1 protein expression. However, the success of any therapeutic strategy depends on the development of economic, effective and efficient methods for miRNAs large-scale production (recombinant production using prokaryotic hosts) and purification, as an alternative to *in vitro* transcription or chemical synthesis^29^. The global interest is to produce high quantities of miRNA but also to obtain and preserve its quality, fulfilling the requirements of regulatory agencies. To accomplish this purpose, we recently described a novel purification methodology based on arginine-affinity chromatography to selectively purify the pre-miR-29b from different RNA species with high recovery yield, purity and good integrity, revealing to be an efficient and reproducible technique to obtain an appropriate RNA quality with potential applicability for transfection studies^29^. Arginine was selected because it is a conserved amino acid of the active center of the PAZ domains of the Argonaute proteins, belonging to the RISC, suggesting that they have an important role in the specific recognition of the 3′UTR of the pre-miRNA^46^. On the other hand, the development of an efficient miRNA delivery system capable to overcome the biological barriers, protect the integrity of miRNA from degradation, promote cellular uptake and, finally, release miRNA in the cytoplasmic compartment is crucial to achieve an improvement in the biological effect in RNAi-based therapies. Hence, according to previous results obtained by our research group^43^, we decided to explore the possibility of using polycations-based carrier systems to efficiently deliver the recombinant pre-miR-29b to N2a695 neuronal cell line, to suppress hBACE1 expression. The polyplexes developed have not a significant cytotoxicity effect of on the selected cell line (Fig. 1). An important *in vitro* experimental approach in this study is the use of a stable N2a695 cell line because in these cells there is a constitutive endogenous hBACE1 expression, enabling greater sensitivity for evaluating the direct or indirect effect of recombinant pre-miR-29b on the cellular levels of hBACE1 or on the products of its activity (Aβ_42_ peptides), once in biological systems over-expressing hBACE1 leads to the formation of Aβ peptides^20^,^44^,^47^.

Along with the role that pre-miR-29b plays in the regulation of the BACE1 levels, it was also evaluated the involvement of this miRNA in the functional regulation of the amyloid pathway. As a matter of fact, in this study and for the first time, it was demonstrated that the overexpression of recombinant pre-miR-29b induces a marked decrease in the levels of the protein hBACE1 and, consequently, a significant decrease in the level of the endogenous Aβ_42_. This result suggests that the application of pre-miR-29 lead to a significant decrease in the levels of two major risk factors commonly associated with the neurodegeneration in AD. We further verified that a single treatment with CS/pre-miR-29b or PEI/pre-miR-29b dramatically decreased the amount of hBACE1 expression (suppressed in 78.5 ± 4.5% and 86.1 ± 1.2%, respectively) and the generation of Aβ_42_ peptide (44.2 ± 6.0% and 47.2 ± 11.4%, respectively) by downregulating hBACE1 protein in N2a695 cells (Figs 2,3 and 5). These findings reinforce the fact that the ability of recombinant pre-miR-29b to regulate endogenous hBACE1 protein expression is likely direct, because it binds to the 3′UTR of hBACE1 mRNA, complementary to the miR-29b seed region (Fig. 4). Our results demonstrated that hBACE1 mRNA levels were also significantly altered following recombinant pre-miR-29b transfection, suggesting a post-transcriptional mechanism that involves direct degradation/destabilization of hBACE1 mRNA and is likely that it promotes inhibition of hBACE1 protein translation. With these results it was verified that hBACE1 is a direct target of pre-miR-29b (Figs 2,3 and 4), what was further validated by the downregulation of hBACE1 gene expression by polycations/pre-miR-29b *in vitro*.

Our findings, together with those from other groups, suggest a fundamental role of miR-29 in AD, and emphasize the potential application of miR-29 in prognosis prediction and AD therapy. With the successful implementation of this methodology and comparing with previously published data (~62% to miR-339-5p^45^; ~35% to miR-29c^21^; ~50% to miR-29a/b-1^16^), we obtained the highest decrease ever reported for the hBACE1 expression levels (around 78% to CS/pre-miR-29b and 86% to PEI/pre-miR-29b) in AD model cells. In addition, it was also verified a decrease of the endogenous Aβ_42_ levels (approximately 44% to CS/pre-miR-29b and 47% to PEI/pre-miR-29b) in these cells. Furthermore, the concentrations of recombinant pre-miR-29b used in this study were much lower than those reported in other works using synthetic miRNA (25 to 250 nM)^16^,^21^, thus a smaller amount of recombinant pre-miR-29b is required to obtain a higher silencing level of hBACE1. In fact, this result may be due to the fact that we are using recombinant pre-miRNA, which is more efficiently recognized and processed within the cell, instead of the mature form of miRNA or the fact that the pre-miRNA was efficiently purified and delivered to the cell through its encapsulation with polycations^48^,^49^. Our results suggest that recombinant pre-miR-29b can represent a novel biopharmaceutical product for the therapeutic modulation of hBACE1 levels, once this study has new implications for hBACE1 biology and offer a new perspective on the treatment of AD. According to the results obtained in this study, we developed an integrative platform that allows biosynthesis, purification and transfection of the recombinant pre-miR-29b using polyplexes to decrease the hBACE1 and endogenous Aβ_42_ expression levels. In addition, this approach allowed improving the currently available methodologies of miRNA-based therapeutics, not only for neurological disorders but also for future therapeutic targets that may be of potential clinical interest.

## Methods

### Reagents

CS (Mw = 50–190 kDa) and cell culture reagents were purchased from Sigma–Aldrich. PEI (Mw = 10 kDa) was purchased from Polysciences. (3-(4,5-dimethylthiazol-2-yl)-5-(3-carboxymethoxyphenyl)-2-(4-sulfophenyl)-2H-tetrazolium) (MTS) was obtained from Promega. Anti-human BACE1 and BACE1 peptide (Abcam); anti-β-actin and monoclonal anti-β-Amyloid (Sigma-Aldrich); Hoechst 33342®, AlexaFluor 488® and AlexaFluor 546® (Invitrogen); Goat anti-rabbit/mouse IgG-HRP (Santa Cruz Biotechnology) were used.

### Pre-miR-29b biosynthesis and purification by arginine affinity chromatography

The pre-miR-29b used in the experiments was produced in a bacterial cell culture of *Rhodovulum sulfidophilum* DSM 1374 strain (BCCM/LMG, Belgium) modified with the plasmid pBHSR1-RM^53^ containing the sequence of pre-miR-29b, as previously described by Pereira and collaborators^33^. Then, the pre-miR-29b isolation was achieved using arginine as a specific ligand in affinity chromatography^33^. The endotoxins content was also evaluated by using the ToxinSensorTM Chromogenic Limulus Amoebocyte Lysate assay kit (GenScript), being verified a considerably reduction during the chromatographic process, since the sample injected onto the arginine matrix had 0.164 EU/μg of sRNA and the final pre-miR-29b fraction presented 0.002 EU/μg of pre-miR-29b. Thus, the endotoxins level in the final pre-miR-29b sample conforms the guidelines of regulatory agencies like Food and Drug Administration (<0.06 EU/mL for the cerebrospinal fluid). In addition, the PCR product corresponding to the purified pre-miR-29b was sequenced to confirm the identity and orientation of the amplicon (Figure S1 in Supporting Information).

### Formulation of polyplexes

Briefly, all the pre-miR-29b loaded polyplexes were formulated using the method of simple complexation which is based on the electrostatic interactions that occur between molar concentrations of positive charge, present in the protonated amine groups of each polycation (N), and the negative charge of the phosphate groups of RNA backbone (P), as described by Pereira and co-workers^41^. The pre-miR-29b (a final concentration of 2 μg/mL) and polycation (a concentration of 10 mg/mL) stock solutions were prepared in sodium acetate buffer (0.1 M sodium acetate/0.1 M acetic acid, pH 4.5). In order to promote encapsulation, cationic polymer solution (100 μL) was added dropwise to the pre-miR-29b solution (400 μL), under stirring during 30 s, to particle formation^41^. The PEI/pre-miR-29b molar ratio is 0.35:1 (mol/mol) and the CS/pre-miR-29b molar ratio is 3:1 (mol/mol). The formulated polyplexes were incubated at RT for 30 min and then pelleted by centrifugation at 15000 g for 20 min. The amount of unbound pre-miR-29b was quantified by UV spectrophotometry^41^. Thus, the encapsulation efficiency (EE) was determined using the following formula: EE% = [(Total pre-miRNA amount – pre-miRNA supernatant amount)/Total pre-miRNA amount] × 100. Three repetitions of this procedure were performed for each system. The hydrodynamic diameter and zeta potential of the pre-miR-29b-loaded polyplexes were determined by dynamic light scattering (DLS) using a Zetasizer Nano ZS particle analyzer (Malvern Instruments, Worcestershire, UK), equipped with a He-Ne laser, at 25 °C. For DLS analysis particle samples were produced as before mentioned and ressuspended in ultrapure water. Size characterization was performed in fully automatic mode and with a scattering angle of 173°. Particle zeta potential measurements were performed in disposable capillary cells and computed by using Henry’s [F(Ka) 1.5], and Smoluchowsky models. All the data were examined in Zetasizer software v 7.03. The experiments were performed in triplicate and an average of 30 measurements was acquired individually for each sample. As a positive control, Lipofectamine 2000 transfection reagent was used (Lipo/pre-miR-29b), according to the protocol recommended by the manufacturer.

### Transfection of N2a695 cells with polyplexes/pre-miR-29b

N2a695 cells at passages 5-27 were cultured in the following medium: 1:1 mixture of Dulbecco’s modified Eagle’s medium (DMEM) and OptiMEM supplemented with 5% (wt/vol) FBS and 1% (wt/vol) penicillin–streptomycin^48^. N2a695 cells were seeded at 3 × 10^4^ cells/mL. When a 50 to 60% confluence was achieved, the culture medium was replaced by serum-free medium. After 12 h, CS/pre-miR-29b, PEI/pre-miR-29b and Lipofectamine/pre-miR-29b (Lipo/pre-miR-29b) were added to the cells at pre-miR-29b concentration of 3.84 to 9.9 nM and transfection was carried out during 6 h. The culture medium was replaced by fresh medium supplemented with 1% FBS and 1% antibiotic, to allow the cells to remain metabolically active, expressing hBACE1 and Aβ. Untreated cells and cells transfected with an unrelated RNA (5′-UGUGCAAAUCUAUGCAAAACUGA-3′) were used for negative controls (at a final concentration of 9.9 nM). In addition, cells were also transfected with scrambled miRNA (5′-UUCUCCGAACGUGUCACGUTT-3′; 3′-TTAAGAGGCUUGCACAGUGCA-5′) and a synthetic miR-29b in the mature form (5′-UAGCACCAUUUGAAAUCAGUGUU-3´) using CS, as controls (at a final concentration of 9.9. nM). The cells were harvested 24, 48 and 72 h after transfection. All transfection experiments were performed in triplicate.

### MTS assay

The cellular cytotoxicity effect of the different formulations of polyplexes/pre-miR-29b was evaluated using the Cell Titer 96® AQueous Non-Radioactive Cell Proliferation Assay. Briefly, N2a695 cells were seeded at a density of 2 × 10^4^ cells per well in a 96-well plate, 24 h after the cell culture medium was replaced by serum-free culture medium. Cells were then transfected as described above and the MTS assay was performed at different time points (48 and 72 h). Subsequently, the medium was exchanged, a mixture of MTS/phenazine metasulfate (PMS) was added to each well, and cells were incubated during 4 h at 37 °C in a humidified atmosphere containing 5% CO_2_. Following incubation, the absorbance measurements of the soluble brown formazan produced were performed in a microplate reader (Sanofi, Diagnostics Pauster) at 492 nm. All experiments were repeated at least three times. Cells incubated with absolute ethanol were used as positive control for cytotoxicity.

### Immunocytochemistry and Imaging Analysis

N2a695 cells were seeded on glass coverslips into 12-well plates, to be recovered at 24, 48 and 72 h. After transfection with either Lipofectamine 2000 or polyplexes, the cells were fixed for 10 min at RT with 4% paraformaldehyde (PFA) buffer solution, followed by permeabilization with 0.1% Triton X-100 for 5 min. Cell preparations were blocked for 1 h at RT with 20% FBS in PBS-T. The cells were then incubated overnight at 4 °C with the primary anti-hBACE1 polyclonal antibody (1:100) and anti-Aβ_17-24_ monoclonal antibody (1:1000). Then, cells were washed 6 times with PBS-T and incubated for 1 h at RT with the appropriate fluorescence conjugated secondary antibodies AlexaFluor 488® goat anti-rabbit or AlexaFluor 546® goat anti-mouse. Lastly, the nucleus were counterstained with Hoechst 33342® (1:1000) for 10 min. Glass coverslips were mounted on slides and the preparations were visualized under a Zeiss LSM 710 laser scanning confocal microscope (Carl Zeiss SMT Inc., USA) equipped with a plane-apocromat 63 × /DIC objective. Images were processed and analyzed using ImageJ software.

### Western blot Analysis

Cells were rinsed in ice-cold PBS and homogenized in cell lysis buffer: 25 mM Tris-HCl buffer, pH 7.4; 2.5 mM EDTA; 1% Triton X-100; 2.5 mM EGTA; 25 mM phenylmethylsulfonyl fluoride and complete EDTA Free protease inhibitor cocktail (Roche). Once homogenized, cell extracts were centrifuged at 11500 rpm for 7 min at 4 °C. The total protein concentration in the supernatant was determined using Bradford Protein Assay (BioRad) and 50 μg of total protein from each cell extract were boiled for 10 min in reducing buffer and then fractionated by electrophoresis on 10% SDS-PAGE. After, proteins were transferred to polyvinylidene difluoride filter (PVDF) membranes and blocked with TBS-T supplemented with 5% BSA. Following, membranes were probed with primary antibodies recognizing the hBACE1 (1:1000 in 5% BSA in TBS-T) and β-actin (1:20000 in TBS-T) at 4 °C overnight. After three washes with TBS-T, membranes were incubated with the HRP-labeled anti-rabbit/mouse IgG secondary antibody diluted 1:50000. Signal detection was performed with ECL substrate (BioRad) according to manufacturer’s instructions and images were acquired with the ChemiDoc™ XRS system (BioRad) and analysed with the Image Lab (BioRad).

### Expression of BACE1 mRNA in N2a695 cells by RT-qPCR

Total RNA was extracted from the cells using TRIzol reagent (Invitrogen). 1 μg of total RNA was reverse transcribed using the RevertAid First Strand cDNA Synthesis Kit (Thermo Fisher Scientific Inc.), according to the manufacturer’s instructions. For quantitative analysis, RT-qPCR amplification of cDNA was performed using the Maxima® SYBR Green/Fluorescein qPCR Master Mix (Thermo Fisher Scientific Inc.) in an IQ5 Cycler from BioRad. RT-qPCR efficiencies were calculated from the given slopes with MyIQ 2.0 software (BioRad). The relative quantification of the BACE1 expression was based on the comparative threshold cycle (C_T_) method in which the amount of the target was determined to be 2^−(ΔCT target−ΔCT calibrator)^, normalized to levels of glyceraldehyde-3-phosphate dehydrogenase (GAPDH) and relative to the untreated control cells. The designed primers for the amplification of hBACE1 and GAPDH are listed in Table S1. Each sample was run in triplicate, and threshold cycle (C_T_) values were averaged from the triplicate. The final data were averaged from 3 separately conducted experiments.

### Sandwich enzyme-linked immunosorbent assay (ELISA)

The endogenous Aβ_42_ levels were quantified in the culture medium and in total cell lysates using ELISA (Invitrogen), according to the manufacturer’s instructions. Total cell lysates were performed as described for Western blot and the culture medium was centrifuged at 13000 rpm for 1 min to remove cell debris. The results were expressed as referred to dilutions of standard synthetic control peptides. In addition, absolute Aβ_42_ values (pg/ml) were measured and normalized to total protein yield of crude cell lysates.

### Statistical analysis

All experiments were repeated at least three times using independent culture preparations. Data in the figures are expressed as mean ± standard error. Quantitative data were statistically analyzed by one-way analysis of variance (ANOVA), followed by pair-wise comparisons using the Fisher’s least significant difference test. * indicate significant difference versus untreated cells, # indicate significant difference versus cells transfected with unrelated RNA control and ● indicate significant difference between samples, being considered statistically significant for p < 0.05. Statistical analysis was performed by using GraphPad Prism 6 software.

## Additional Information

**How to cite this article**: Pereira, P. A. *et al*. Recombinant pre-miR-29b for Alzheimer´s disease therapeutics. *Sci. Rep.* **6**, 19946; doi: 10.1038/srep19946 (2016).

## Supplementary Material

Supplementary Information

## Figures and Tables

**Table 1:** Characterization of polyplexes.

**Polyplexes**	**N/P Ratio**	**Size (nm)**	**Zeta potential (mV)**	**Encapsulation efficiency**
Chitosan	30	130.65 ± 8.29	+ 24.53 ± 2.92	90%
Polyethylenimine	3.5	118.92 ± 5.10	+ 35.00 ± 4.22	100%

The values of zeta potential and size were calculated with the data obtained from three independent measurements (mean ± SD, n = 3).

## References

[b1] Broderick J. A. & Zamore P. D. MicroRNA therapeutics. Gene Ther 18, 1104–10 (2011). 21525952 10.1038/gt.2011.50 PMC3237828

[b2] Bartels C. L. & Tsongalis G. J. MicroRNAs: novel biomarkers for human cancer. Clin Chem 55, 623–31 (2009). 19246618 10.1373/clinchem.2008.112805

[b3] Selbach M. . Widespread changes in protein synthesis induced by microRNAs. Nature 455, 58–63 (2008). 18668040 10.1038/nature07228

[b4] Ambros V. The functions of animal microRNAs. Nature 431, 350–5 (2004). 15372042 10.1038/nature02871

[b5] He L. & Hannon G. J. MicroRNAs: small RNAs with a big role in gene regulation. Nat Rev Genet 5, 522–31 (2004). 15211354 10.1038/nrg1379

[b6] Bartel D. P. MicroRNAs: genomics, biogenesis, mechanism, and function. Cell 116, 281–97 (2004). 14744438 10.1016/s0092-8674(04)00045-5

[b7] Bartel D. P. MicroRNAs: target recognition and regulatory functions. Cell 136, 215–33 (2009). 19167326 10.1016/j.cell.2009.01.002 PMC3794896

[b8] Chen F. & Hu S. J. Effect of microRNA-34a in cell cycle, differentiation, and apoptosis: a review. J Biochem Mol Toxic 26, 79–86 (2012). 10.1002/jbt.20412 22162084

[b9] Plaisier C. L., Pan M. & Baliga N. S. A miRNA-regulatory network explains how dysregulated miRNAs perturb oncogenic processes across diverse cancers. Genome Res 22, 2302–14 (2012). 22745231 10.1101/gr.133991.111 PMC3483559

[b10] Jiang H., Zhang G., Wu J. H. & Jiang C. P. Diverse roles of miR-29 in cancer (review). Oncol Rep 31, 1509–16 (2014) 24573597 10.3892/or.2014.3036

[b11] Wu Z., Huang X., Zou Q. & Guo Y. The inhibitory role of Mir-29 in growth of breast cancer cells. J Exp Clin Canc Res 32, 98 (2013). 10.1186/1756-9966-32-98 PMC4176287 24289849

[b12] Xiong Y. . Effects of microRNA-29 on apoptosis, tumorigenicity, and prognosis of hepatocellular carcinoma. Hepatology 51, 836–45 (2010). 20041405 10.1002/hep.23380

[b13] Li Y. . Progressive miRNA expression profiles in cervical carcinogenesis and identification of HPV-related target genes for miR-29. J Pathol 224, 484–95 (2011). 21503900 10.1002/path.2873

[b14] Fabbri M. . MicroRNA-29 family reverts aberrant methylation in lung cancer by targeting DNA methyltransferases 3A and 3B. Proc Natl Acad Sci USA 104, 15805–10 (2007). 17890317 10.1073/pnas.0707628104 PMC2000384

[b15] Jiao J., Herl L. D., Farese R. V. & Gao F. B. MicroRNA-29b regulates the expression level of human progranulin, a secreted glycoprotein implicated in frontotemporal dementia. PloS One 5, e10551 (2010). 20479936 10.1371/journal.pone.0010551 PMC2866661

[b16] Hebert S. S. . Loss of microRNA cluster miR-29a/b-1 in sporadic Alzheimer’s disease correlates with increased BACE1/beta-secretase expression. Proc Natl Acad Sci USA 105, 6415–20 (2008). 18434550 10.1073/pnas.0710263105 PMC2359789

[b17] Satoh J. MicroRNAs and their therapeutic potential for human diseases: aberrant microRNA expression in Alzheimer’s disease brains. J Pharmacol Sci 114, 269–75 (2010). 20953120 10.1254/jphs.10r11fm

[b18] Kocerha J., Kauppinen S. & Wahlestedt C. microRNAs in CNS disorders. Neuromolecular Med 11, 162–72 (2009). 19536656 10.1007/s12017-009-8066-1

[b19] Hebert S. S. . MicroRNA regulation of Alzheimer’s Amyloid precursor protein expression. Neurobiol Dis 33, 422–8 (2009). 19110058 10.1016/j.nbd.2008.11.009

[b20] Bettens K. . APP and BACE1 miRNA genetic variability has no major role in risk for Alzheimer disease. Hum Mutat 30, 1207–13 (2009). 19462468 10.1002/humu.21027

[b21] Zong Y. . miR-29c regulates BACE1 protein expression. Brain Res 1395, 108–15 (2011). 21565331 10.1016/j.brainres.2011.04.035

[b22] Dominguez D. I., Hartmann D. & De Strooper B. BACE1 and presenilin: two unusual aspartyl proteases involved in Alzheimer’s disease. Neurodegener Dis 1, 168–74 (2004). 16908986 10.1159/000080982

[b23] Cole S. L. & Vassar R. The Alzheimer’s disease beta-secretase enzyme, BACE1. Mol Neurodegener 2, 22 (2007). 18005427 10.1186/1750-1326-2-22 PMC2211305

[b24] Schonrock N., Matamales M., Ittner L. M. & Gotz J. MicroRNA networks surrounding APP and amyloid-beta metabolism-implications for Alzheimer’s disease. Exp Neurol 235, 447–454 (2012). 22119426 10.1016/j.expneurol.2011.11.013

[b25] Hunt C. E. & Turner A. J. Cell biology, regulation and inhibition of beta-secretase (BACE-1). FEBS J 276, 1845–59 (2009). 19292866 10.1111/j.1742-4658.2009.06929.x

[b26] Ponchon L. & Dardel F. Large scale expression and purification of recombinant RNA in Escherichia coli. Methods 54, 267–73 (2011). 21320602 10.1016/j.ymeth.2011.02.007

[b27] Sherlin L. D. . Chemical and enzymatic synthesis of tRNAs for high-throughput crystallization. RNA 7, 1671–8 (2001). 11720294 PMC1370207

[b28] Martins R., Queiroz J. A. & Sousa F. Ribonucleic acid purification. J Chromatogr A 1355, 1–14 (2014) 24951289 10.1016/j.chroma.2014.05.075

[b29] Pereira P. . Purification of pre-miR-29 by arginine-affinity chromatography. J Chromatogr B Analyt Technol Biomed Life Sci 951–952, 16–23 (2014). 10.1016/j.jchromb.2014.01.020 24508674

[b30] Pecot C. V., Calin G. A., Coleman R. L., Lopez-Berestein G. & Sood A. K. RNA interference in the clinic: challenges and future directions. Nat Rev Cancer 11, 59–67 (2011). 21160526 10.1038/nrc2966 PMC3199132

[b31] Lee S. Y. . Stability and cellular uptake of polymerized siRNA (poly-siRNA)/polyethylenimine (PEI) complexes for efficient gene silencing. J Control Release 141, 339–46 (2010). 19836427 10.1016/j.jconrel.2009.10.007

[b32] Nimesh S. & Chandra R. Polyethylenimine nanoparticles as an efficient *in vitro* siRNA delivery system. Eur J Pharm Biopharm 73, 43–9 (2009). 19362592 10.1016/j.ejpb.2009.04.001

[b33] Kwok A. & Hart S. L. Comparative structural and functional studies of nanoparticle formulations for DNA and siRNA delivery. Nanomed-Nanotechnol 7, 210–9 (2011). 10.1016/j.nano.2010.07.005 20709624

[b34] Grayson A. C., Doody A. M. & Putnam D. Biophysical and structural characterization of polyethylenimine-mediated siRNA delivery *in vitro*. Pharmaceutical research 23, 1868–76 (2006). 16845585 10.1007/s11095-006-9009-2

[b35] Lungwitz U., Breunig M., Blunk T. & Gopferich A. Polyethylenimine-based non-viral gene delivery systems. Eur J Pharm Biopharm 60, 247–66 (2005). 15939236 10.1016/j.ejpb.2004.11.011

[b36] Sarvaiya J. & Agrawal Y. K. Chitosan as a suitable nanocarrier material for anti-Alzheimer drug delivery. Int J Biol Macromol 72, 454–65 (2015). 25199867 10.1016/j.ijbiomac.2014.08.052

[b37] Howard K. A. . RNA interference *in vitro* and *in vivo* using a novel chitosan/siRNA nanoparticle system. Mol Ther 14, 476–84 (2006). 16829204 10.1016/j.ymthe.2006.04.010

[b38] Holzerny P. . Biophysical properties of chitosan/siRNA polyplexes: profiling the polymer/siRNA interactions and bioactivity. J Control Release 157, 297–304 (2012). 21884740 10.1016/j.jconrel.2011.08.023

[b39] Rudzinski W. E. & Aminabhavi T. M. Chitosan as a carrier for targeted delivery of small interfering RNA. Int J Pharm 399, 1–11 (2010). 20732398 10.1016/j.ijpharm.2010.08.022

[b40] Mao S., Sun, W., Kissel, T. Chitosan-based formulations for delivery of DNA and siRNA. Adv Drug Deliver Rev 62, 12–27 (2010). 10.1016/j.addr.2009.08.004 19796660

[b41] Pereira P. . Characterization of polyplexes involving small RNA. J Colloid Interface Sci 387, 84–94 (2012). 22980740 10.1016/j.jcis.2012.07.088

[b42] Godbey W. T., Wu K. K. & Mikos A. G. Size matters: molecular weight affects the efficiency of poly(ethylenimine) as a gene delivery vehicle. J Biomed Mater Res 45, 268–75 (1999). 10397985 10.1002/(sici)1097-4636(19990605)45:3<268::aid-jbm15>3.0.co;2-q

[b43] Godbey W. T., Wu K. K. & Mikos A. G. Poly(ethylenimine) and its role in gene delivery. J Control Release 60, 149–60 (1999). 10425321 10.1016/s0168-3659(99)00090-5

[b44] Chen Y. . Antidiabetic drug metformin (GlucophageR) increases biogenesis of Alzheimer’s amyloid peptides via up-regulating BACE1 transcription. Proc Natl Acad Sci USA 106, 3907–12 (2009). 19237574 10.1073/pnas.0807991106 PMC2656178

[b45] Long J. M., Ray B. & Lahiri D. K. MicroRNA-339-5p down-regulates protein expression of beta-site amyloid precursor protein-cleaving enzyme 1 (BACE1) in human primary brain cultures and is reduced in brain tissue specimens of Alzheimer disease subjects. J Biol Chem 289, 5184–98 (2014). 24352696 10.1074/jbc.M113.518241 PMC3931075

[b46] Rivas F. V. . Purified Argonaute2 and an siRNA form recombinant human RISC. Nat Struct Mol Biol 12, 340–9 (2005). 15800637 10.1038/nsmb918

[b47] Kobayashi D. . BACE1 gene deletion: impact on behavioral function in a model of Alzheimer’s disease. Neurobiol Aging 29, 861–73 (2008). 17331621 10.1016/j.neurobiolaging.2007.01.002

[b48] Tsutsumi A., Kawamata T., Izumi N., Seitz H. & Tomari Y. Recognition of the pre-miRNA structure by Drosophila Dicer-1. Nat Struct Mol Biol 18, 1153–8 (2011). 21926993 10.1038/nsmb.2125

[b49] Li M. M., Wang W. P., Wu W. J., Huang M. & Yu A. M. Rapid production of novel pre-microRNA agent has-mir-27b in *Escherichia coli* using recombinant RNA technology for functional studies in mammmalian cells. Drug Metab Dispos 42, 1791–1794 (2014). 25161167 10.1124/dmd.114.060145 PMC4201134

