# Supplementary material for: Recombinant pre-miR-29b for Alzheimer´s disease therapeutics
Source: Sci Rep. 2016 Jan 28;6:19946. doi: 10.1038/srep19946 (PMC4730146; doi:10.1038/srep19946)
Supplement: Supplementary Information [file srep19946-s1.doc]

**Supporting Information**

**Recombinant pre-miR-29b for Alzheimer´s disease therapeutics**

Patrícia A. Pereira*, Joana F. Tomás*, João A. Queiroz*, Ana R. Figueiras*^,#^, Fani Sousa*

^*^CICS-UBI – Health Sciences Research Centre, University of Beira Interior, Avenida Infante D. Henrique, Covilhã, 6200-506, Portugal; ^#^CNC – Center of Neuroscience and Cell Biology, University of Coimbra, Largo Marquês de Pombal, Coimbra, 3004-517, Portugal

**Figure S1** - Sequencing of the pre-miR-29b following the protocol GenomeLabTM Dye Terminator Cycle Sequencing with Quick Start Kit for GenomeLabTM GeXP sequencer (Beckman Coulter). Sequence data was analyzed using GenomeLab system Beckman Coulter version 10.2 software.


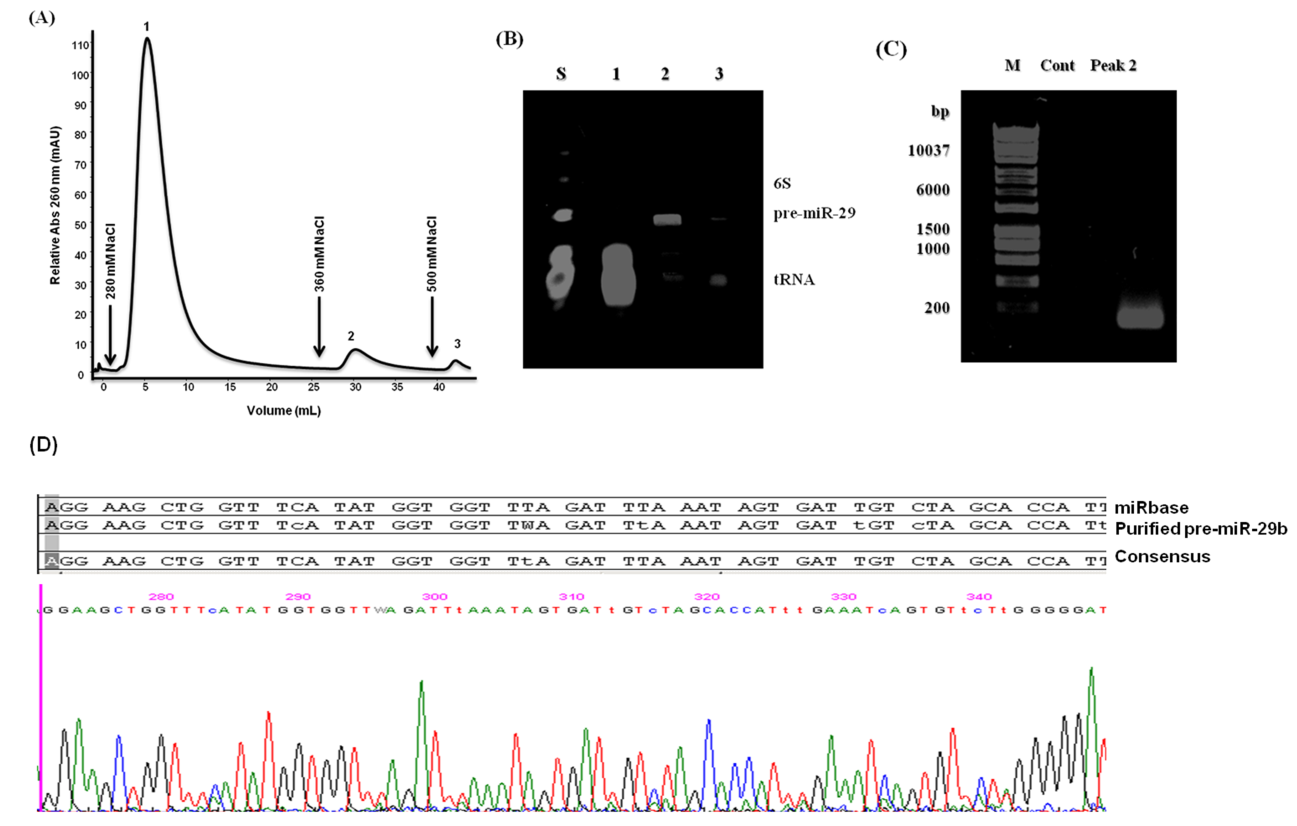


**Table S1** – Primers used for the amplification of hBACE1 and GAPDH.

| **Primer** | **Forward** | **Reverse** |
| --- | --- | --- |
| hBACE1 | 5’-AGACGCTCAACATCCTGGTG-3’ | 5’-CCTGGGTGTAGGGCACATAC-3’ |
| GAPDH | 5’-TGACGTGCCGCCTGGAGAAA-3’ | 5’-AGTGTAGCCCAAGATGCCCTTCAG-3’ |
